# Optimizing the Graphene/α-Al_2_O_3_(0001) Interface through Minimization of Interfacial Stress for Improved Electronic Applications

**DOI:** 10.1021/acsanm.5c03894

**Published:** 2025-11-18

**Authors:** Debdipto Acharya, Daniele Perilli, Cristiana Di Valentin

**Affiliations:** Department of Materials Science, 9305 University of Milano-Bicocca, via R. Cozzi 55, 20125 Milano, Italy

**Keywords:** graphene, density functional theory (DFT) calculations, Al_2_O_3_, electronics, insulating substrate, buckling

## Abstract

The direct integration of graphene onto technologically relevant insulating substrates is crucial for next-generation electronic and optoelectronic devices. Here, we present a density functional theory (DFT) study of the structural, electronic, and adhesion properties of graphene on the α-Al_2_O_3_(0001) surface. A 12-layer Al-terminated slab with two middle layers fixed is shown to provide an optimal balance between computational efficiency and accuracy, reproducing key surface properties such as work functions and electronic structures. The adsorption of graphene reveals a transition from planar to corrugated geometries with an increasing supercell size. Buckling notably modifies the local electronic structure, inducing a small bandgap and charge redistribution within graphene without significant charge transfer to the substrate. Energetics, corrugation patterns, and simulated scanning tunnelling microscopy images indicate that the interaction is dominated by weak van der Waals forces and lattice-induced modulation. Additionally, a rotated (R30) graphene configuration minimizes interface strain and exhibits enhanced stability. These findings offer valuable insights into the interfacial physics of graphene on dielectric oxides, relevant for applications in electronics, optoelectronics, and sensing.

## Graphical abstract

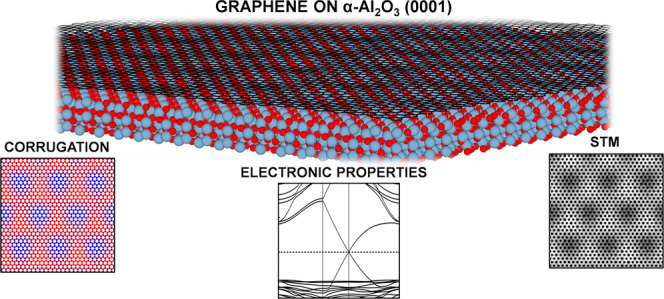

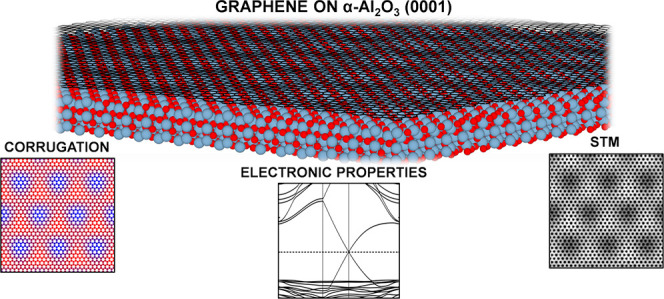

## 1 Introduction

Interfacing graphene (Gr) with other materials is an effective strategy to tune its physical and chemical properties for applications including catalysis, electronics, and gas sensing.^1–4^ Transition metals have been widely employed as catalysts for Gr growth via chemical vapor deposition (CVD), and the choice of metal substrate critically determines the strength of the Gr–metal interaction.^5,6^ On weakly interacting substrates such as Al(111), Pt(111), and Cu(111), Gr largely retains its intrinsic linear Dirac dispersion, with only minor charge transfer or orbital hybridization.^7^ In contrast, strong π–d hybridization on Ni(111) and Co(0001) suppresses the Dirac cone and markedly alters Gr’s electronic structure.^7,8^ Medium- to strongly interacting metals like Ir(111)^9,10^ and Ru(0001)^11^ can also induce morphological changes, including lattice corrugation and Moiré pattern formation. The interaction strength – ranging from physisorption to chemisorption – governs key properties such as doping and band gap opening, making Gr/metal interfaces versatile platforms for interface engineering and heterostructure design.

Despite extensive research on Gr/metal systems, metals are not ideal for many practical applications. Devices such as field-effect transistors (FETs), chemiresistors, photodetectors, and flexible transparent electronics require Gr to be supported on insulating substrates.^12^ Typically, CVD-grown Gr is transferred from metal to insulator, but these steps introduce defects and contaminants and increase fabrication cost and complexity. To overcome these issues, direct CVD growth of Gr on insulating metal oxides has gained increasing attention.^13–17^ Although these materials are less catalytically active than metals, they enable direct integration of Gr onto functional insulating supports, offering a promising route toward transparent, scalable, and high-performance Gr-based electronics.^18–20^ However, achieving wafer-scale, single-crystal Gr directly on dielectrics remains challenging, as the limited catalytic activity and surface uniformity of substrates such as SiO_2_,^13^ hexagonal boron nitride (h-BN),^21^ TiO_2_,^22,23^ and glass^14^ often yield polycrystalline films with misaligned domains and grain boundaries.

Among metal oxides, sapphire (α-Al_2_O_3_) – and particularly its most stable (0001) surface – has attracted considerable interest due to its potential for direct integration of Gr on insulating substrates.^15,16,24–32^ In the past decade, several studies have successfully demonstrated the direct CVD growth of continuous, wafer-scale monolayer Gr on Al_2_O_3_(0001) (Scheme 1).^16,17^ However, this surface poses fundamental challenges. Despite being extensively investigated for decades, its structure and composition remain not fully understood. The Al_2_O_3_(0001) surface exhibits a complex morphology, with various terminations and reconstructions depending on preparation methods and environmental conditions. Freshly etched or ultrahigh vacuum (UHV) annealed surfaces typically display a (1 × 1) pattern^33^ associated with an unreconstructed Al-terminated surface,^34^ also observed after thermal treatment in air.^35^ This agrees with DFT results showing that O-terminated surfaces are less stable than Al-terminated ones under both oxygen-rich and oxygen-poor environments.^31,36^ At higher annealing temperatures (1300–1700 K), a more complex (√31 × √31)*R* ± 9° reconstruction forms,^37,38^ while exposure to water or hydrogen stabilizes O-terminated surfaces through hydroxylation, yielding Gibbsite-like terminations.^39,40^

**Scheme 1:**
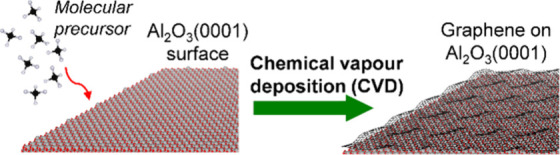
Schematic Representation of the CVD Growth of Gr on Al_2_O_3_(0001)

This diversity of surface terminations directly affects the structure of the Gr/Al_2_O_3_(0001) interface. For example, thermal CVD growth can induce complex reconstructions: Dou et al.,^41^ combining aberration-corrected transmission electron microscopy (Cs-TEM) and DFT, showed that Gr grown on an initially Al-terminated surface ultimately contacts O atoms formed during high-temperature growth. Annealing in H_2_ before CVD can instead promote the (√31 × √31)*R* ± 9° reconstruction, as observed by Mishra et al.^16^ via LEED. Wördenweber et al.^30^ proposed an alternative interface in which Gr interacts with a hydroxylated alumina surface, leading to a band gap opening of approximately (73 ± 3) meV. In contrast, plasma-enhanced CVD (PECVD) allows low-temperature growth that can avoid reconstruction and preserve the unreconstructed substrate.^31^ Overall, no single Gr/Al_2_O_3_(0001) interface exists; its structure depends strongly on both the growth conditions and the initial substrate state.

Despite this recent experimental progress, the theoretical understanding of the Gr/Al_2_O_3_(0001) interface remains limited. Only a few computational studies have investigated its structural and electronic properties,^15,17,31,41–46^ and even fewer have addressed how these depend on the size and orientation of Gr domains.^15^ Key aspects such as lattice mismatch, surface-induced corrugation, and interfacial electronic coupling are still not well understood. Previous calculations^17,31,41–46^ that we are aware of have employed commensurate models that artificially align Gr and Al_2_O_3_, resulting in compressed Gr layers or stretched oxide slabs and restricting the analysis to small supercells unable to capture strain relaxation. These modeling choices can bias predictions of charge transfer, π–d hybridization, and modifications of Gr’s electronic structure, issues that must be resolved to enable rational interface design for devices that require large-area Gr on wide-band gap dielectrics.

In this work, we aim to fill this knowledge gap by conducting a comprehensive first-principles investigation using dispersion-corrected density functional theory (DFT) to explore the structural and electronic interactions at the Gr/Al_2_O_3_(0001) interface. We systematically investigate both planar and corrugated Gr configurations, examining the effects of cell size and lattice orientation between Gr and Al_2_O_3_(0001) on the structural and electronic properties, and analyze the resulting charge redistribution and band structure modifications induced by Gr adsorption. Unlike previous studies, we employ large supercell models containing up to ∼1800 atoms to accurately capture the interfacial interactions. In addition, an energy decomposition analysis is performed to break down the various contributions to the interfacial interaction. Overall, this study provides critical insights into the complex interplay between strain, surface morphology, and electronic properties at the graphene/oxide interface, offering guidance for future experimental developments in electronic and optoelectronic device fabrication.

## 2 Computational Methods

We performed ab initio density functional theory (DFT) calculations as implemented in the Quantum ESPRESSO package.^47,48^ The exchange-correlation interactions were described using the generalized gradient approximation (GGA) of the Perdew–Burke–Ernzerhof (PBE) form.^49^ The interaction between ionic cores and valence electrons was described using ultrasoft pseudopotential.^50^ Electronic wave functions were expanded using a plane wave basis set. We tested the convergence of wave function and charge density cutoffs for bulk α-Al_2_O_3_ (30 atoms), obtaining final values of 45 and 450 Ry, respectively. *k*-point convergence was also verified by testing several Monkhorst–Pack^51^ meshes with respect to the total energy and electronic band gap (Figure S1). Based on these tests, a 12 × 12 × 5 *k*-point grid was selected for the conventional hexagonal unit cell and proportionally reduced meshes were used for larger supercells. Dispersion interactions were included using the DFT+D3 scheme.^52^ Marzari-Vanderbilt cold smearing^53^ with a width of 0.001 Ry was used to aid convergence.

For slab calculations, a 15.5 Å vacuum was added along the surface normal to avoid interactions between periodic images, and a dipole correction was applied.^54^ Monkhorst–Pack k-point meshes were chosen according to the supercell size (Section 3). For the (6 × 6)/(3 × 3) Gr/Al_2_O_3_(0001) cell, 4 × 4 × 1 and 12 × 12 × 1 meshes were used for geometry relaxation and electronic structure calculations, respectively. For the (12 × 12)/(6 × 6) Gr/Al_2_O_3_(0001) cell, 1 × 1 × 1 (Γ-centered) and 2 × 2 × 1 meshes were used for geometry optimization and electronic structure analysis, respectively. For the (9√3 × 9√3)/(8 × 8) Gr/Al_2_O_3_(0001) cell, a Γ-centered 1 × 1 × 1 mesh was used for both geometry optimization and electronic structure analysis due to the high computational cost of the model (containing 1766 atoms).

The adsorption of Gr on Al_2_O_3_(0001) surface is defined as

$$\Delta E_{\mathrm{ads}}=E_{\mathrm{Gr}/{\mathrm{Al}}_{2}O_{3}}-(E_{{\mathrm{Al}}_{2}O_{3}}+E_{\mathrm{Gr}})$$ 1

where *E*_Gr/Al_2_O_3__ is the total energy of the optimized Gr/Al_2_O_3_(0001) system, *E*_Al_2_O_3__ is the energy of the relaxed Al_2_O_3_ surface, and *E*_Gr_ is the energy of a fully relaxed (cell and atomic positions), flat Gr sheet consisting of *n* atoms.

The corrugation energy is defined as

$$\Delta E_{\mathrm{corr}}=E_{\mathrm{Gr}-\mathrm{corr},{\mathrm{Al}}_{2}O_{3}-\mathrm{lattice}}-E_{\mathrm{Gr}-\mathrm{plan},{\mathrm{Al}}_{2}O_{3}-\mathrm{lattice}}$$ 2

where *E*_Gr–corr,Al_2_O_3_–lattice_ is the total energy of the optimized corrugated Gr layer constrained to the Al_2_O_3_ lattice parameter, and *E*_Gr–plan,Al_2_O_3_–lattice_ is the energy of the corresponding optimized planar Gr layer under the same lattice compression.

As detailed in Section 3.2.5, and following a similar approach adopted in some of our previous works,^55,56^ we further decomposed Δ*E*_ads_ to gain deeper insight into the energy contributions governing the Gr/Al_2_O_3_ interaction. Specifically, Δ*E*_ads_ was separated into three components:

i) the Gr compression energy (Δ*E*_comp,Gr_);
ii) the deformation energy of both Gr and Al_2_O_3_ upon interaction (Δ*E*_def_);
iii) the adhesion energy of Gr on Al_2_O_3_ (Δ*E*_adh_).

These components are defined as follows:

$$\Delta E_{\mathrm{comp},\mathrm{Gr}}=E_{\mathrm{Gr},\mathrm{compressed}}-E_{\mathrm{Gr}}$$ 3

where *E*_Gr, compressed_ is the total energy of a planar Gr sheet, compressed to match the Al_2_O_3_ cell and containing *n* atoms, and *E*_Gr_ is the energy of a fully relaxed, flat Gr sheet with the same number of atoms. To ensure a fair comparison between these two systems, the out-of-plane (*z*) coordinate of the compressed cell is adjusted to match the volume of the relaxed graphene sheet.

$$\Delta E_{\mathrm{def}}=(E_{\mathrm{Gr},\mathrm{Gr}/{\mathrm{Al}}_{2}O_{3}}-E_{\mathrm{Gr},\mathrm{compressed}})+(E_{{\mathrm{Al}}_{2}O_{3},\mathrm{Gr}/{\mathrm{Al}}_{2}O_{3}}-E_{{\mathrm{Al}}_{2}O_{3}})$$ 4

where *E*_Gr, Gr/Al_2_O_3__ and *E*_Al_2_O_3_, Gr/Al_2_O_3__ are the total energies of the Gr and Al_2_O_3_ components, respectively, extracted from the relaxed Gr/Al_2_O_3_ interface geometry. These values represent the energies of the individual components in their deformed, interacting states.

$$\Delta E_{\mathrm{adh}}=E_{\mathrm{Gr}/{\mathrm{Al}}_{2}O_{3}}-(E_{{\mathrm{Al}}_{2}O_{3},\mathrm{Gr}/{\mathrm{Al}}_{2}O_{3}}+E_{\mathrm{Gr},\mathrm{Gr}/{\mathrm{Al}}_{2}O_{3}})$$ 5

To compute the atomic charges, partitioning the electronic charge density is done according to the Bader charge analysis.^57^

The Climbing Image–Nudged Elastic Band (CI–NEB) method^58^ was employed to simulate the energy barrier between planar and corrugated graphene. STM simulations were performed using the Tersoff-Hamann approach,^59^ according to which the tunnelling current is proportional to the energy-integrated Local Density of States (ILDOS).

### 2.1 Preliminary Calculations: Bulk α-Al_2_O_3_

We first performed DFT calculations to optimize the structure of bulk α-Al_2_O_3_. The lattice parameters and internal atomic positions were relaxed, yielding an in-plane lattice constant of 4.79 Å and a *c*-axis length of 13.06 Å. These values are in good agreement with previously reported data obtained using various exchange–correlation functionals,^60,61^ as well as with experimental measurements, as summarized in Table S1 of the Supporting Information.

The electronic band structure and projected density of states (PDOS) of bulk α-Al_2_O_3_ are shown in Figure S2. The calculated band gap is 6.02 eV and is direct at the Γ point. The valence band is mainly composed of O 2p orbitals, with minor contributions from Al 3p and Al 3s states. The conduction band minimum, characterized by a low density of states, primarily arises from Al 3s, O 2s, and Al 3p orbitals.

Although the band gap computed using the PBE functional is underestimated – a known limitation of standard DFT – calculations using hybrid functionals yield values much closer to experiment, namely 8.62 eV with HSE06 and 9.38 eV with PBE0, compared to the experimental value of 8.8 eV.^62^ Our results are in excellent agreement with previously reported band gaps calculated using different functionals, as summarized in Table S1 of the Supporting Information.

## 3 Results and Discussion

### 3.1 Al-Terminated Al_2_O_3_ (0001) Surface

Both experimental and theoretical studies have consistently shown that the unreconstructed α-Al_2_O_3_(0001) surface is Al-terminated (AlO_3_Al-R, where R denotes the continuation of the bulk stacking sequence), identifying it as the most stable and commonly observed termination under a wide range of environmental conditions. For instance, surface phase diagrams computed from DFT calculations indicate that the Al-terminated surface is more stable than the O-terminated one across a broad range of oxygen chemical potentials, from O-poor to O-rich regimes.^31,36^ Experimental observations obtained by heating Al_2_O_3_(0001) in air further confirm this conclusion.^33–35^ Owing to its well-established structural and energetic stability, we adopt this configuration as the reference model for our investigation. Earlier computational works employed an 18-layer Al-terminated α-Al_2_O_3_(0001) slab, relaxing the top 11 layers while fixing the bottom 7, to study adsorption of atomic oxygen, nitrogen, and carbon monoxide.^60,63^ In the present study, we re-examine this model to determine whether a thinner slab can reproduce the essential surface properties with comparable accuracy. To this end, we systematically compare Al-terminated α- Al_2_O_3_(0001) slabs of varying thicknesses.

By comparing Al_2_O_3_(0001) slabs consisting of 12, 15, and 18 atomic layers, each with different combinations of fixed and relaxed layers, we found that a 12-layer slab with the two central layers kept fixed offers a suitable balance between computational efficiency and accuracy in surface properties (Figure 1a; see also Figure S3 for additional structural details). The calculated work function for the 12-layer slab is 6.46 eV, closely matching the 6.47 eV obtained for the fully relaxed 18-layer slab. Figure 1b,c shows the computed band structure and projected density of states (PDOS) for the 12-layer slab, while Figure S4 compares the corresponding results for the 12-layer (two fixed middle layers), 15-layer (fully relaxed), and 18-layer (fully relaxed) slabs. The results reveal nearly identical electronic structures, with only minor differences in the band gap values (4.52 eV for the 12-layer slab, 4.57 eV for the 15-layer slab, and 4.62 eV for the 18-layer slab). These findings validate the 12-layer Al-terminated α-Al_2_O_3_(0001) slab as a reliable and computationally efficient model for all subsequent Gr adsorption simulations.

**Figure 1:**
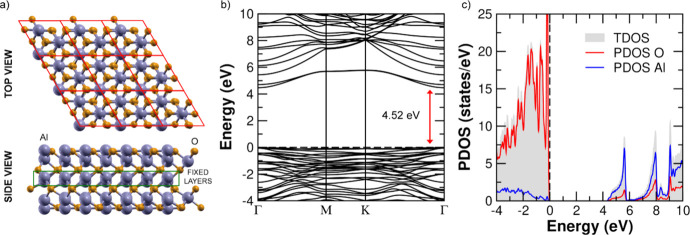
(a) Top and side views of the optimized 12-layer Al-terminated Al_2_O_3_(0001) surface. The unit cell, outlined in red in the top view, is replicated three times along both periodic directions. (b) PBE+D3 band structure and (c) PDOS of the Al_2_O_3_(0001) surface. The PDOS legend is shown in panel (c). All energies are referenced to the valence band maximum (set to 0 eV). The direct band gap at the Γ point is marked by a red arrow in panel (b).

### 3.2 Graphene on the Al_2_O_3_(0001) Surface

#### 3.2.1 Flat Graphene on the Al_2_O_3_ Surface

According to previous theoretical studies,^15,17,31,41–46^ the smallest commensurate for Gr domain is built by aligning the Gr and Al_2_O_3_ lattice vectors (R0), which results in a lattice mismatch of about 2.95% and requires compressing the Gr lattice to match the substrate. To investigate the adsorption behavior, we considered several stacking configurations of Gr on a 12-layer Al_2_O_3_(0001) surface (Figure 2). These configurations differ in the relative registry between C atoms in Gr and Al/O atoms in the topmost substrate layer, as highlighted by the red circles in Figure 2. Among the considered structures, the most stable corresponds to the configuration where one C atom in Gr lies directly above a surface Al atom, as shown in Figure 2a**.**

**Figure 2:**
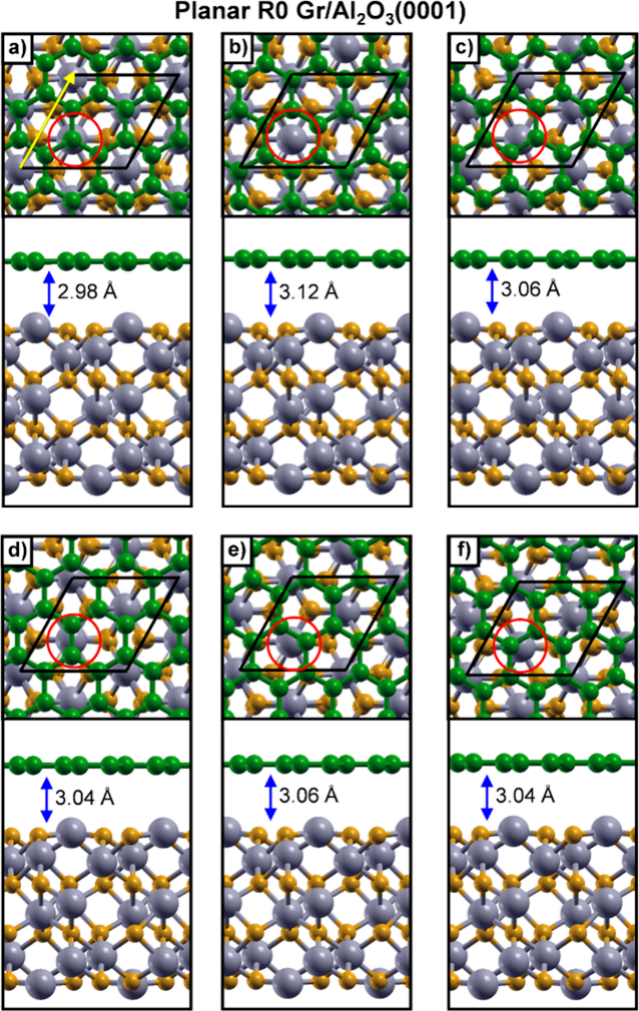
Top and side views of planar R0 (2 × 2) Gr on a (1 × 1) 12-layer Al_2_O_3_(0001) slab with different stacking configurations (a–f). The unit cell is outlined in black. Panel (a) shows the lowest-energy configuration. (Å). Color scheme: Al (gray), O (dark yellow), and C (green). Red circles mark surface Al atoms, and the yellow arrow in (a) indicates the side-view direction. The Gr– Al_2_O_3_ distance, indicated by a blue arrow, is reported in each panel.

After structural relaxation, Gr remains flat, with a vertical distance of 2.98 Å from the Al_2_O_3_(0001) surface. Notably, both the interlayer distance and the adsorption energy of this configuration are in good agreement with previous results obtained using projector augmented-wave (PAW) pseudopotentials.^45^ A detailed comparison of relative energies and graphene–Al_2_O_3_ vertical distances for all tested configurations is summarized in Table S2. The energy differences across the various stacking arrangements are minimal and closely match the reference values obtained using a fully relaxed 18-layer Al_2_O_3_ slab, also included in Table S2, further validating the use of the 12-layer model.

The electronic band structure (Figure 3a) and PDOS (Figure 3b) of the lowest-energy configuration show that the Dirac point of Gr lies at the Fermi level, with no charge transfer to the Al_2_O_3_ substrate, as confirmed by computed atomic charges. This indicates that Gr remains largely decoupled from the substrate, preserving its intrinsic electronic properties.

**Figure 3:**
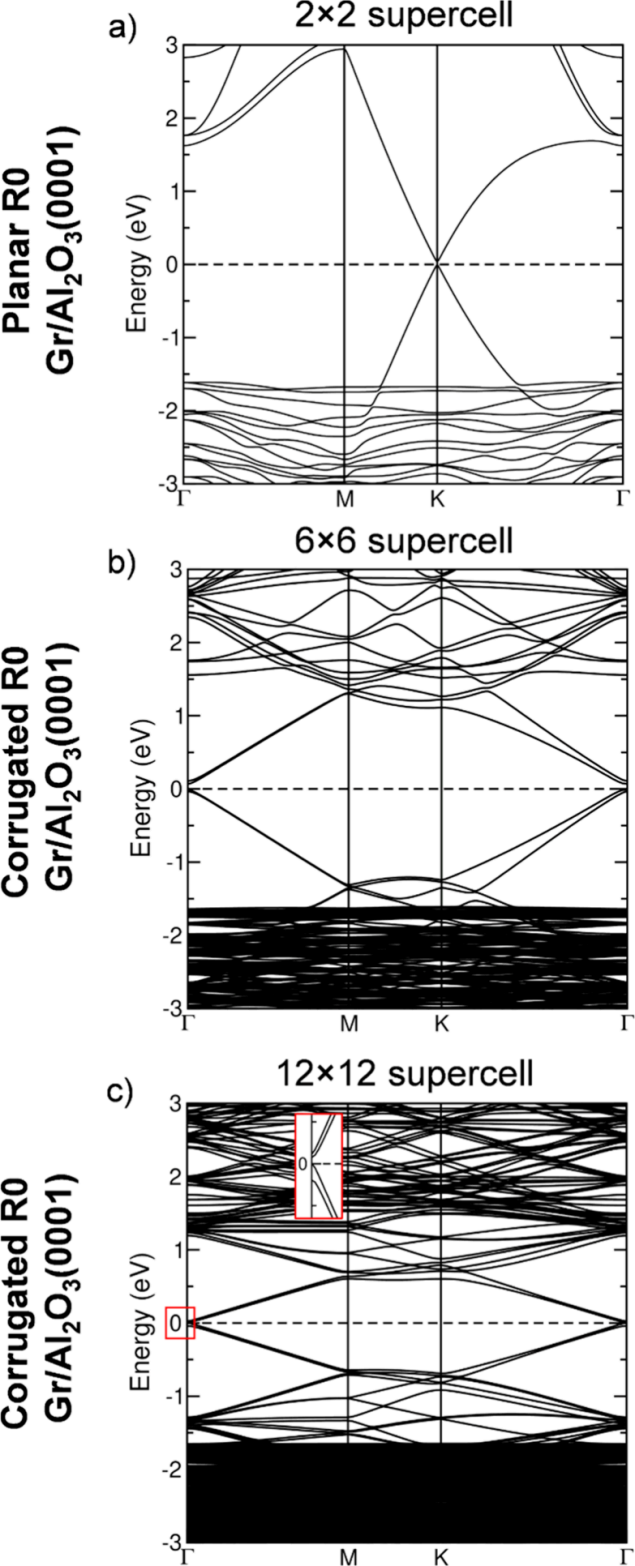
Band structures of the most stable (a) planar R0 (2 × 2) (Figure 2a), (b) corrugated R0 (6 × 6) (Figure 4a), and (c) R0 (12 × 12) (Figure 6a) Gr/Al_2_O_3_(0001) interfaces. All energies are referenced to the Fermi level, set to 0 eV. The inset in panel (c) highlights the band gap opening. For the (6 × 6) and (12 × 12) models, the Dirac cone is located at Γ.

#### 3.2.2 Corrugated Graphene on the Al_2_O_3_(0001) Surface

As a next step, we investigated the adsorption of Gr on the 12-layer Al_2_O_3_ (0001) surface using a larger supercell. Specifically, a (6 × 6) Gr sheet was placed on a (3 × 3) Al_2_O_3_(0001) cell, maintaining the same 2.95% lattice mismatch as in the (2 × 2) Gr on (1 × 1) Al_2_O_3_ case discussed above. In contrast to the smaller system, where Gr likely remains artificially planar due to the constraints of the limited cell size and periodic boundary conditions, the larger periodic sheet exhibits noticeable corrugation upon relaxation (Figure 4). As with the planar case, we tested several stacking arrangements for corrugated Gr. The lowest-energy configuration is the one in which rows of low-lying C atoms align directly above rows of surface Al atoms, as shown in Figure 4a.

**Figure 4:**
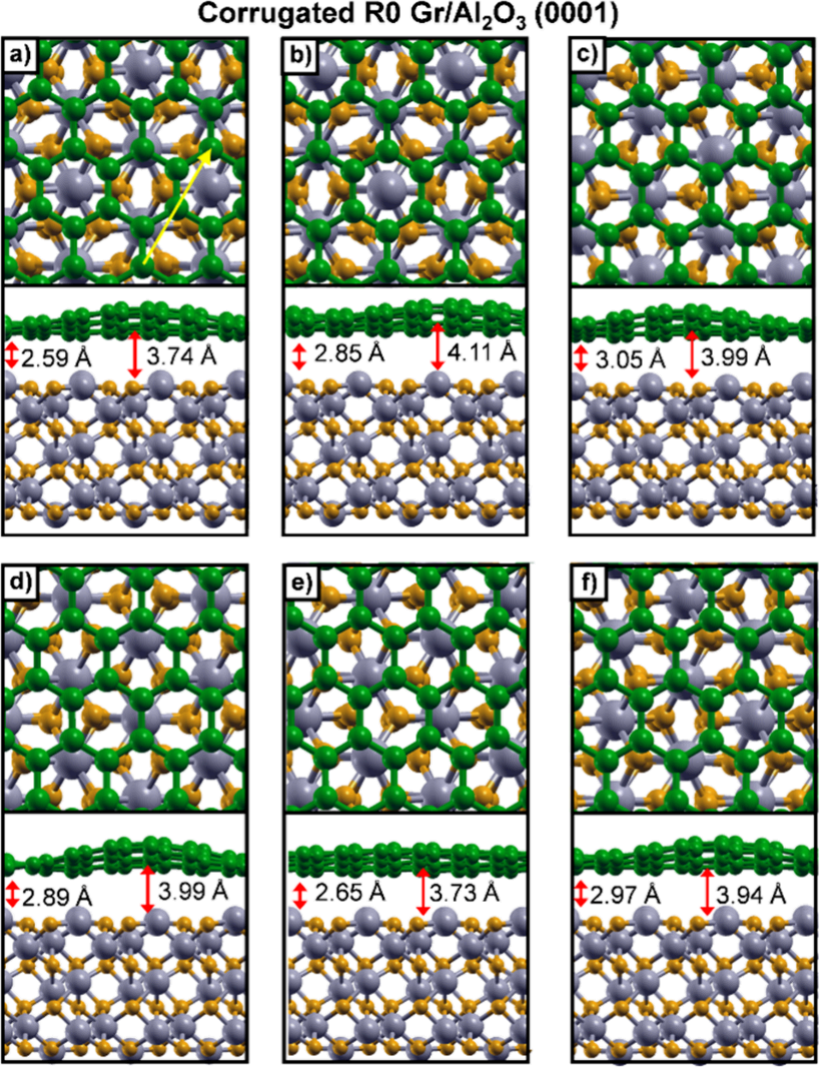
Top and side views of corrugated R0 (6 × 6) Gr on a (3 × 3) 12-layer Al_2_O_3_(0001) slab with different stacking configurations (a–f). Panel (a) shows the lowest-energy configuration. The shortest and largest Gr– Al_2_O_3_ distances (Å) are provided in each panel. For clarity, only part of the (6 × 6) Gr cell is displayed to highlight the stacking with the substrate. Color scheme: Al (gray), O (dark yellow), and C (green). The yellow arrow in (a) indicates the side-view direction.

For the lowest energy configuration (Figure 4a), the vertical distance between Gr and the Al_2_O_3_ surface in the corrugated structure ranges from 2.59 and 3.74 Å, with an average value of 3.09 Å, compared to 2.9 Å for the planar configuration discussed in the previous section. The calculated relative energies for the various stacking configurations of corrugated Gr on the 12-layer Al_2_O_3_(0001) surface are summarized in Table S3.

Similar to the planar case, the energy differences among the various stacking configurations of corrugated Gr are minimal. To better understand the stability of planar graphene on the Al_2_O_3_ surface, as observed both in this work and in previous studies,^45^ we performed a CI-NEB calculation to estimate the energy barrier between the planar and corrugated configurations.

In this calculation, we considered a planar R0 (6 × 6) Gr sheet and a corrugated R0 (6 × 6) Gr sheet adsorbed on a (3 × 3) Al_2_O_3_(0001) surface. The resulting energy profile, shown in Figure S5a, shows the transition pathway from the planar to the corrugated configuration. Despite including several intermediate climbing images, the barrier is found to be negligible (2 meV), indicating that the transition is kinetically accessible at room temperature. A clearer view of the barrier is provided by zooming into the initial segment of the CI-NEB path, as shown in the inset of Figure S5a. These results confirm that the planar graphene configuration represents a local minimum on the potential energy surface.

This is the first report of corrugated Gr on the Al_2_O_3_(0001) surface. To assess how corrugation affects in-plane mobility, we investigated the sliding behavior of both planar and corrugated Gr on the Al_2_O_3_ surface. For each case, two key configurations were considered: one with a C atom directly above a surface Al atom (Figures 2a and 4a) and one with a C atom aligned with a surface O atom (Figures 2c and 4c). Figure S5b shows that the energy barrier for planar Gr is very low (0.04 eV), indicating facile in-plane motion. In contrast, corrugated Gr exhibits a substantially higher barrier (≈0.6 eV, Figure S5c), suggesting that corrugation strongly hinders lateral sliding and makes movement across the surface energetically less favorable.

In Figure 3b, we report the band structure of corrugated R0 Gr on Al_2_O_3_(0001), corresponding to the configuration in Figure 4a. Unlike in the planar case, where the Dirac cone is preserved at the Fermi level, the corrugated R0 (6 × 6) Gr exhibits a band gap opening of ≈80 meV, indicating modification of its electronic structure due to corrugation. It should be noted that our calculations employ the PBE+D3 functional, which tends to underestimate band gaps; therefore, the discussion is intended to highlight qualitative rather than quantitative trends.^64^ Nonetheless, Bader charge analysis confirms that no significant charge transfer occurs between Gr and the substrate.

Although no net charge transfer occurs between Gr and the Al_2_O_3_(0001) surface, corrugation induces a charge redistribution within the Gr layer. As shown in Figure S6, charge accumulates in the lower regions, closer to the substrate, where negatively charged C atoms interact electrostatically with the positively charged surface Al atoms, while charge depletion occurs in the upper regions, farther from the surface.

To gain deeper insight into the corrugation pattern, we visualized the vertical displacement of carbon atoms using a color gradient. In Figure 5a, the local height of Gr is represented as a heatmap: red denotes the geometrical highest atoms, while blue indicates the geometrical lowest. The gradient between these extremes reflects the variation in corrugation across the surface. Notably, the vertical stripes of blue, indicating regions of minimum height, align with the underlying array of surface aluminum atoms, suggesting that the Gr corrugation is guided by the atomic arrangement of the Al_2_O_3_ substrate.

**Figure 5:**
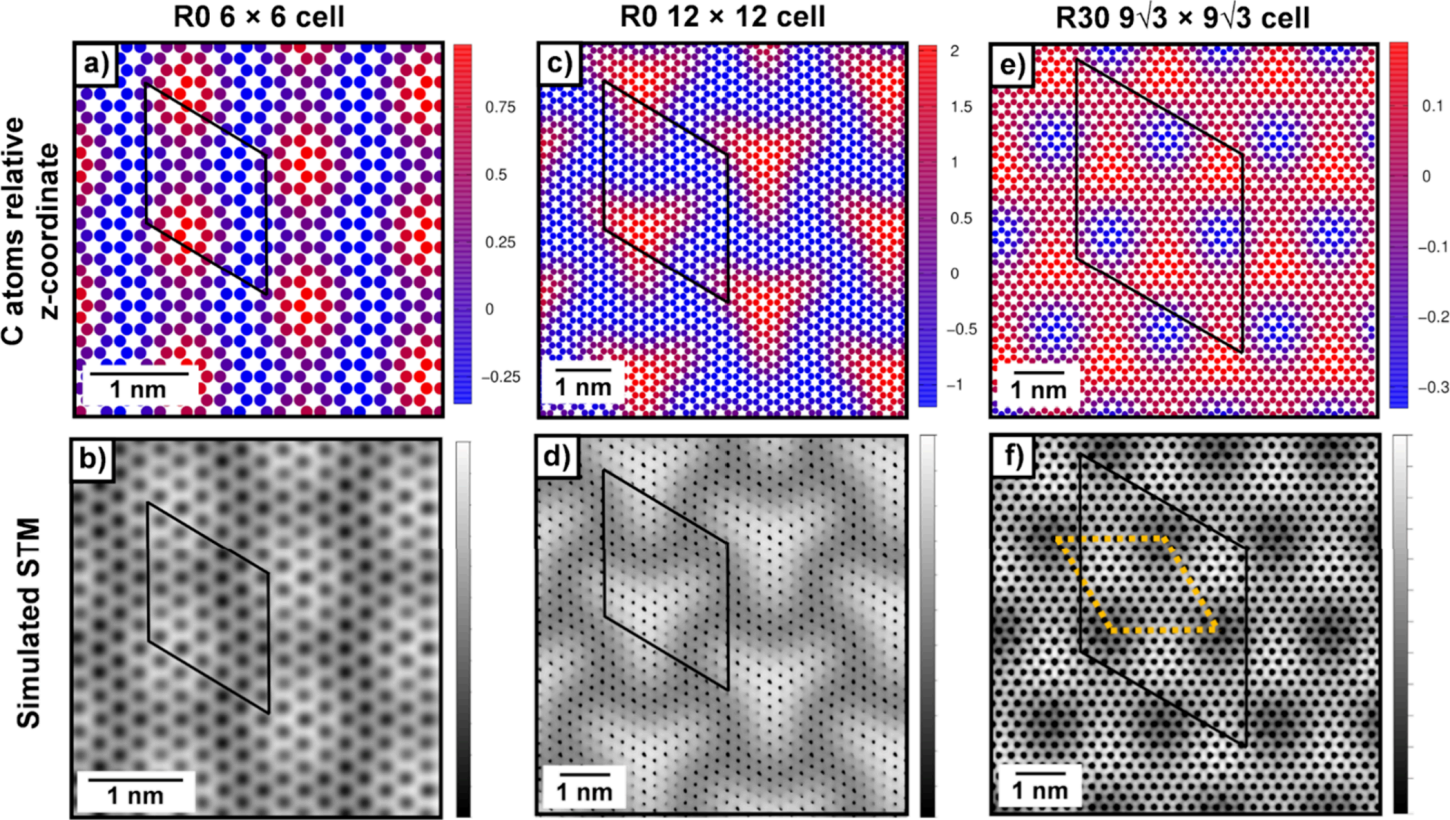
Vertical corrugation of graphene in the (a) R0 (6 × 6), (c) R0 (12 × 12), and (e) R30 (9√3 × 9√3) Gr/Al_2_O_3_(0001) models, shown as color maps (different scales enhance contrast). Red and blue indicate the highest and lowest C atoms, respectively. Heights are given relative to the average C *z*-coordinate (Å). Panels (b), (d), and (f) show the corresponding simulated STM images, whose contrast follows the corrugation pattern in (a). Bias voltages of 0.5, 1.0, and 0.6 V were used in (b), (d), and (f). The Gr supercell is outlined in black. The yellow cell in panel (b) highlights the smallest STM periodic pattern for the R30 model.

To further support this interpretation, we simulated the STM image of the corrugated Gr on the Al_2_O_3_(0001) surface, as shown in Figure 5b. The resulting STM contrast closely follows the local topography of the Gr layer, indicating that the apparent brightness is primarily governed by the height of carbon atoms.

#### 3.2.3 Corrugated Graphene on the Al_2_O_3_ (0001) Surface with an Increasing Supercell Size

Since Gr tends to corrugate upon adsorption on the Al_2_O_3_(0001) surface, we next investigated how the supercell size influences the extent of this corrugation. To this end, we examined a larger system composed of a R0 (12 × 12) Gr supercell on a (6 × 6) Al_2_O_3_(0001) surface supercell. Although the lattice mismatch remains identical (2.95%) as in the smaller systems ((2 × 2) Gr on (1 × 1) Al_2_O_3_ and (6 × 6) Gr on (3 × 3) Al_2_O_3_), the large cell provides more degrees of freedom for relaxation. The optimized top and side views of the corrugated Gr are shown in Figure 6a. Here, the vertical distance between Gr and the Al_2_O_3_ substrate ranges from 2.58 to 5.73 Å, with an average value of 3.66 Å. These values are significantly larger than those observed for the smaller (6 × 6) Gr on (3 × 3) Al_2_O_3_ system (2.59–3.74 Å, average 3.09 Å) and for the (2 × 2) planar Gr on (1 × 1) Al_2_O_3_ system (2.90 Å).

**Figure 6:**
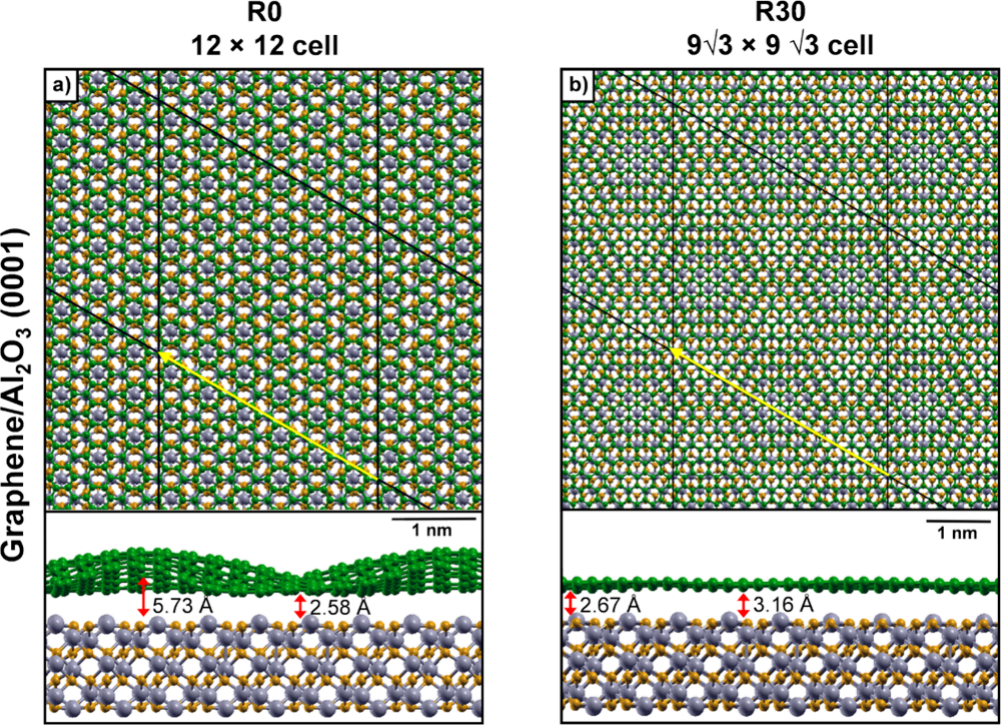
Top and side views of (a) corrugated R0 (12 × 12) graphene on a 12-layer (6 × 6) Al_2_O_3_(0001) surface and (b) R30 (9√3 × 9√3) graphene. Color scheme: Al (gray), O (dark yellow), and C (green). Yellow arrows in panels (a) and (b) indicate the viewing direction for the corresponding side views shown below. To better highlight Gr corrugation, the side views are periodically replicated along the *xy* plane. The shortest and largest vertical distances between graphene and the Al_2_O_3_ surface are given in Å.

We then analyzed the electronic structure of the larger supercell, with the corresponding band structure shown in Figure 3b. As in the smaller systems, a band gap opening of ≈17 meV is observed for the R0 (12 × 12) graphene on the (6 × 6) Al_2_O_3_ surface. Notably, despite the pronounced corrugation, no net charge transfer is observed between Gr and the substrate.

The spatial distribution of Gr corrugation in the larger supercell is illustrated in Figure 5c using a color gradient map, analogous to that shown for the smaller cell. A comparison between Figure 5a,c reveals that increasing the supercell size not only amplifies the corrugation amplitude but also modifies its overall shape. The simulated STM image in Figure 5d closely matches the corrugation pattern observed in the color map, further confirming that the STM contrast is directly determined by the Gr height modulation (compare Figure 5c,d).

Since the corrugation pattern depends on the chosen supercell model, it is important to characterize it quantitatively. We therefore introduce two metrics describing the corrugation amplitude and its spatial periodicity.

The first metric, *h*_corr_, is the root-mean-square (RMS) deviation of the Gr *z*-coordinates, defined as

$$h_{\mathrm{corr}}=\sqrt{\frac{1}{N}\sum_{i=1}^{N}(z_{i}-z_{\mathrm{av}}{)}^{2}}$$ 6

where *N* is the total number of Gr atoms, *z*_*i*_ the *z*-coordinate of the *i*-th atom, and *z*_av_ the average z-coordinate. A larger *h*_cor_ value indicates a higher corrugation amplitude.

The second metric, denoted as λ, represents the spatial periodicity (wavelength) of the corrugation. For the R0 supercells, corrugation follows a periodic pattern defined by the lattice vectors; thus, λ corresponds to the lattice periodicity of the respective supercell. The calculated *h*_cor_ and λ values for the R0 models are summarized in Table 1.

**Table 1:** Summary of the Corrugation Amplitude (*h*_corr_), Spatial periodicity (λ), and Corresponding Electronic Bandgap Values for the R0 and R30 Gr/Al_2_O_3_(0001) Interface Models^a^

model	*h*_corr_ (Å)	λ (Å)	bandgap (meV)
R0 (6 × 6)	0.39	14.4	80
R0 (12 × 12)	0.92	28.7	17
R30 (9√3 × 9√3)	0.13	22.1	

a No band gap value was computed for the R30 configuration due to the high computational cost.

The 6 × 6 supercell exhibits a smaller corrugation amplitude and a shorter wavelength than the 12 × 12 one. This may also explain the smaller band gap in the larger system: although the corrugation amplitude increases (0.92 Å vs 0.39 Å), the deformation varies more smoothly (period of 28.7 Å vs 14.4 Å), resulting in a weaker perturbation of the π-symmetry. Thus, a larger corrugation amplitude does not necessarily correspond to a stronger local lattice distortion governing the band gap opening.

Finally, to investigate the origin of this corrugation, we performed test calculations in which free-standing Gr was fully relaxed while its lattice parameters were fixed to those of Al_2_O_3_(0001), imposing a 2.95% compressive strain. Both planar and corrugated configurations were obtained, with the latter being more stable by −0.86 eV (−12 meV per C atom), confirming that compressed Gr intrinsically tends to buckle. However, the resulting corrugation pattern differs from that of the supported system, highlighting that the substrate plays a crucial role in defining the specific corrugation geometry.

#### 3.2.4 Rotated Gr (R30) on the Al_2_O_3_(0001) Surface

Experimental studies using Low-Energy Electron Diffraction (LEED) have confirmed that Gr predominantly aligns at a 30° angle relative to the Al_2_O_3_(0001) surface.^15,16^ However, previous theoretical investigations^15^ have modeled this system using finite Gr clusters (e.g., C_24_H_12_), which may not fully capture the effects of an extended interface. To address this limitation, we constructed a commensurate supercell (containing more than 1700 atoms) consisting of a (9√3 × 9√3) Gr cell rotated by 30° (R30) on an (8 × 8) Al_2_O_3_(0001) surface cell. This configuration yields a much lower lattice mismatch of only 0.31% (compressive), compared to 2.95% in unrotated models. Table S4 summarizes the smallest commensurate supercells that can be generated for different allowed rotational angles between Gr and Al_2_O_3_(0001), while keeping the lattice mismatch below ±1%. The results demonstrate that the R30 model provides the lowest mismatch with the fewest atoms.

Figure 6b shows the optimized top and side views of R30 Gr on the Al_2_O_3_ surface. Despite the minimal strain, the Gr layer exhibits slight corrugation, with vertical distances from the substrate ranging from 2.67 to 3.16 Å (average distance of 2.97 Å), smaller than the range observed for the unrotated (6 × 6) and (12 × 12) Gr supercells. The corrugation pattern of R30 Gr is illustrated using a color gradient in Figure 5e. The amplitude of the corrugation (*h*_cor_) is significantly lower compared to the strained supercells (Table 1), namely (6 × 6) Gr on (3 × 3) Al_2_O_3_ and (12 × 12) Gr on (6 × 6) Al_2_O_3_.

Interestingly, while the corrugation patterns in the (6 × 6) and (12 × 12) Gr configurations exhibit periodicities that match their respective supercell sizes, the R30 graphene displays a distinct corrugation periodicity (λ) of ≈22.1 Å, different from its supercell dimension of 38.29 Å. We investigated the possibility of constructing a smaller supercell that matches the 22.1 Å periodicity, however, we realized it is not possible to build a Al_2_O_3_ surface cell with this periodicity.

Finally, we simulated the STM image of R30 graphene on the Al_2_O_3_(0001) surface (Figure 5f). The contrast closely follows the corrugation pattern of the Gr layer (compare Figure 5e,f), confirming that STM contrast in this case is also primarily governed by geometric effects. Overall, the R30 configuration, with its minimal strain, reduced corrugation, and alignment with experimental observations, offers a realistic model of Gr on Al_2_O_3_(0001).

#### 3.2.5 Energy Decomposition Analysis

To gain deeper insight into the thermodynamic origins of Gr corrugation on the Al_2_O_3_ surface, we performed a detailed energy decomposition analysis for four representative systems: R0 (6 × 6) planar, R0 (6 × 6) corrugated, R0 (12 × 12) corrugated, and R30 (9√3 × 9√3) Gr. As a first step, we calculated the strain-corrected corrugation energy of free-standing Gr compressed to match the substrate, thereby assessing how strain and supercell size affect the energetic cost of corrugation (Table 2). This energy is defined as the difference between corrugated, optimized free-standing compressed Gr and planar Gr in its fully relaxed (unstrained) unit cell, thus accounting for both the energy penalty of compression and the stabilization gained through corrugation.

**Table 2:** Strain-Corrected Corrugation Energies of Free-Standing Gr Calculated for R0 (6 × 6), R0 (12 × 12), and R30 (9√3 × 9√3) Supercells Relative to Planar Gr^a^

Gr rotational angle	free standing system	strain-corrected corrugation energy (meV/C)
	Gr planar	0
R0	(6 × 6) Gr corrugated	+51.2
R0	(12 × 12) Gr corrugated	+28.7
R30	(9√3 × 9√3) Gr	+2.5

a All atomic positions were fully relaxed. Planar Gr is optimized in its lattice constant, while corrugated and R30 structures were matched to the Al_2_O_3_(0001) lattice. The unit cell volume was kept constant by adjusting the *z*-coordinate to allow direct energy comparison.

As summarized in Table 3, the (6 × 6) corrugated Gr exhibits a strain-corrected corrugation energy of +51.2 meV per carbon atom. In contrast, the larger (12 × 12) system yields a substantially lower value of +28.7 meV per carbon atom. This decrease indicates that larger supercells provide greater configurational freedom, allowing Gr to adopt lower-energy corrugated structures. Meanwhile, the R30 Gr, subject to only 0.31% compressive strain, exhibits a nearly negligible strain-corrected corrugation energy of just +2.5 meV per carbon atom. These results underscore the central role of lattice strain and mismatch in driving corrugation in Gr on Al_2_O_3_.

**Table 3:** Energy Contributions Obtained from the Decomposition Analysis, As Illustrated in Figure 7, for the Different Graphene/Al_2_O_3_(0001) Interface Models^a^

	Δ*E*_ads_ (eV)	Δ*E*_compr,Gr_ (eV)	Δ*E*_def_ (eV)	Δ*E*_corr_ (meV/C)	Δ*E*_adh_ (eV)
R0 (6 × 6) Gr planar 72 C atoms 180 Al/O atoms	+0.887	+4.595	+0.066 (+0.018 + 0.048)	0	–3.774
	+0.012 per C	+0.064 per C			–0.052 per C
R0 (6 × 6) Gr corrugated 72 C atoms 180 Al/O atoms	+0.389	+4.595	–0.561 (−0.794 + 0.233)	–11	–3.645
	+0.005 per C	+0.064 per C			–0.051 per C
R0 (12 × 12) Gr corrugated 288 C atoms 720 Al/O atoms	–1.536	+18.380	–8.771 (−9.495 + 0.724)	–33	–11.145
	–0.005 per C	+0.064 per C			–0.039 per C
R30 (9√3 × 9√3) R30 Gr 486 C atoms 1280 Al/O atoms	–24.881	+0.640	+0.888 (+0.253 + 0.634)	+0.5	–26.409
	–0.051 per C	+0.001 per C			–0.054 per C

a Reported values include the graphene compression energy (Δ*E*_compr,Gr_), the total deformation energy (Δ*E*_def_), with individual contributions from graphene and the Al_2_O_3_ surface indicated in parentheses, the adhesion energy (Δ*E*_adh_), and the resulting total adsorption energy (Δ*E*_ads_). All energies are given in eV.

To further analyze the adsorption energetics, we decomposed the total adsorption energy into three main components: compression energy, deformation energy, and adhesion energy. This decomposition is illustrated schematically in Figure 7, with detailed numerical values reported in Table 3. By isolating these individual contributions, we can better understand how different factors influence the binding strength and stability of graphene on the Al_2_O_3_ surface.

**Figure 7:**
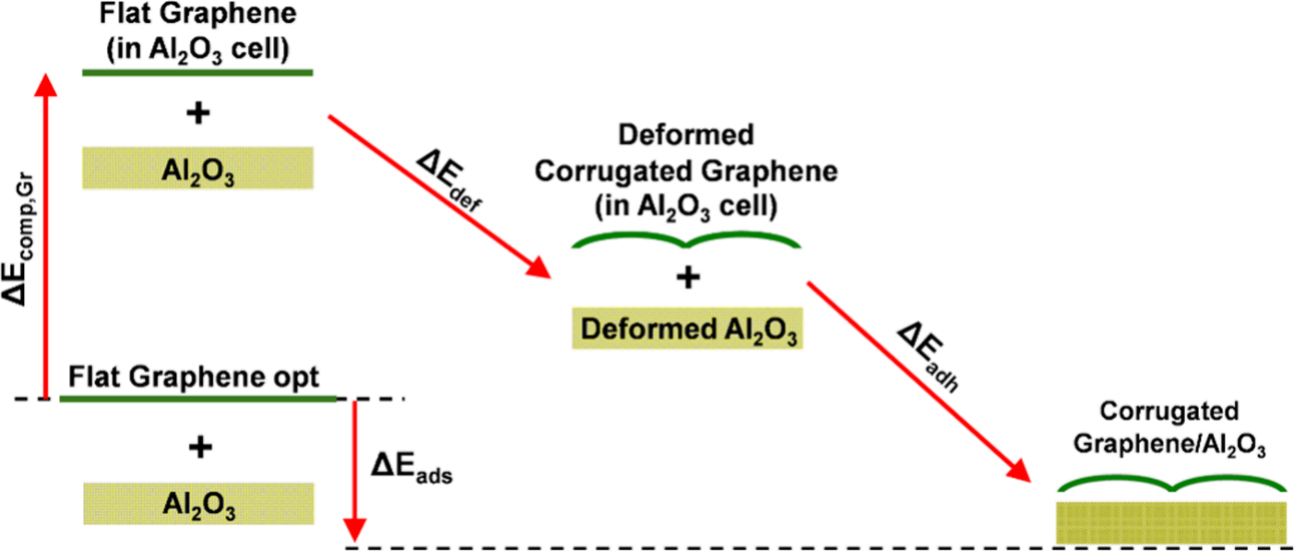
Schematic representation of the energy decomposition analysis for graphene adsorption on Al_2_O_3_(0001). The total adsorption energy (Δ*E*_a__d__s_, negative) is divided into Gr compression (Δ*E*_compr,Gr_, positive), total deformation (Δ*E*_def_, positive), and adhesion (Δ*E*_adh_, negative) terms. The deformation energy includes separate contributions from Gr and the substrate. Corresponding values are reported in Table 3 and computed as described in Section 2.

The adsorption energy (Δ*E*_ads_) is defined as the difference between the total energy of the Gr/Al_2_O_3_ system and the sum of the energies of its fully relaxed, isolated components, planar Gr and the Al_2_O_3_ slab. This value reflects the overall strength of the interaction between the Gr layer and the substrate.

The compression energy (Δ*E*_compr,Gr_) quantifies the energetic penalty required to compress the Gr lattice to match the periodicity of the Al_2_O_3_ surface. The deformation energy (Δ*E*_def_) accounts for the energy cost (or gain) associated with structural distortions: specifically, the transformation of the compressed planar Gr into its adsorbed geometry, along with any deformation of the Al_2_O_3_ surface itself. We also evaluate the corrugation energy (Δ*E*_corr,Gr_), defined as the energy required to deform the compressed planar graphene into a corrugated configuration, reported per carbon atom.

Lastly, the adhesion energy (Δ*E*_adh_) represents the stabilizing interaction between the deformed Gr and the Al_2_O_3_ substrate. Based on the laws of thermodynamical cycles, the total adsorption energy equals the sum of the compression, deformation, and adhesion energies: Δ*E*_ads_ = Δ*E*_compr,Gr_ + Δ*E*_def_ + Δ*E*_adh_.

Table 3 summarizes the calculated adsorption, compression, deformation, corrugation, and adhesion energies for four interface models: 6 × 6 planar Gr, 6 × 6 corrugated Gr, 12 × 12 corrugated Gr, and R30 Gr on the Al_2_O_3_ (0001) surface.

We start by analyzing the adsorption energies. Notably, only the (12 × 12) corrugated Gr (5 meV/C) and R30 Gr (51 meV/C) configurations exhibit negative adsorption energies on the Al_2_O_3_ (0001) surface, indicating thermodynamically favorable binding. Adsorption energies were computed using flat Gr in its equilibrium lattice as a reference, i.e., without deformation. In the R0 (6 × 6) model, the positive adsorption energy results from compressive strain in Gr upon adsorption, not compensated by substrate interaction, unlike in the R0 (12 × 12) model. Under experimental conditions, however, such systems are typically grown by CVD, where the reference state involves molecular precursors rather than preformed Gr. Therefore, the calculated adsorption energies are most meaningful for comparing different models, and a positive value does not necessarily imply that the corresponding structure cannot exist experimentally. The origin of these trends becomes evident from the energy decomposition analysis.

For all systems except R30 Gr, the compression energy required to match the Al_2_O_3_ lattice is substantial, amounting to approximately 64 meV per carbon atom. In contrast, the deformation energy associated with the Al_2_O_3_ slab remains relatively small across all models. The deformation energy of Gr, equivalent to the corrugation energy per carbon atom, is zero for planar Gr and negligible (2.5 meV/C) for R30 Gr, but increases significantly in strained systems, reaching 11 meV/C for the (6 × 6) and 33 meV/C for the (12 × 12) corrugated Gr.

The adhesion energy, reflecting the stabilizing interaction between deformed Gr and the substrate, is relatively uniform across most systems, ranging from 51 to 54 meV/C. The exception is the (12 × 12) corrugated model, where the adhesion energy decreases to 39 meV/C due to the increased average vertical distance between Gr and the Al_2_O_3_ surface, which weakens interfacial interactions.

In summary, the pronounced negative adsorption energy of R30 Gr stems primarily from its minimal compression and deformation energy penalties, rather than from a substantially stronger adhesion to the substrate compared to the other models.

## 4 Conclusions

In this study, we performed an in-depth dispersion-corrected DFT (PBE+D3) analysis of the structural, electronic and adhesion properties of Gr on α-Al_2_O_3_(0001) surface. After benchmarking different slab models, we determined that a 12-layer slab of α-Al_2_O_3_(0001) with two middle layers fixed provides an optimal balance between computational efficiency and accuracy, faithfully reproducing key surface properties such as the work function and electronic structure.

We systematically investigated the adsorption of both planar and corrugated Gr on the Al_2_O_3_(0001) surface. While planar Gr preserves its characteristic Dirac cone – indicating weak interaction with the substrate – larger supercells exhibit spontaneous corrugation of the Gr layer. The corrugated configurations show a small band gap (≈80 meV at the PBE+D3 level) at the Dirac point and charge redistribution within the Gr sheet, although no significant charge transfer occurs across the interface.

Increasing the Gr supercell size enhances the corrugation amplitude, keeping a band gap opening (≈17 meV at the PBE+D3 level) and distinct simulated STM patterns. Finally, by introducing a 30° rotation of the Gr layer (R30 configuration), we identified a nearly strain-free commensurate interface with significantly reduced corrugation energy and strong adsorption. This configuration aligns with experimental observations and offers the most energetically stable geometry among all considered systems.

Overall, our findings elucidate the complex interplay of lattice strain, surface corrugation, and electronic structure at the Gr/Al_2_O_3_ interface, offering critical insights for the design of high-performance Gr-based electronic and optoelectronic devices.

## Supplementary Material



## Notes

The benchmark of exchange–correlation functionals (GGA and hybrid) for bulk α-Al_2_O_3_; comparing lattice parameters and band gaps with literature values; energetics and Gr–Al_2_O_3_(0001) distances for R0 (2 × 2) and (6 × 6) interfaces; lattice mismatches and structural details for commensurate Gr/α- Al_2_O_3_(0001) models at different rotations; *k*-point convergence tests for bulk α- Al_2_O_3_; band structures and PDOS for the optimized bulk; the model of the 12-layer Al_2_O_3_(0001) slab; band structures and PDOS for slabs of different thicknesses; CI-NEB energy profiles for Gr corrugation and sliding on Al_2_O_3_(0001); and atomic charge plot for the corrugated R0 Gr (6 × 6) model (PDF)
