# Supplementary material for: Optimizing the Graphene/α-Al2O3(0001) Interface through Minimization of Interfacial Stress for Improved Electronic Applications
Source: ACS Appl Nano Mater. 2025 Nov 18;8(47):22626–36. doi: 10.1021/acsanm.5c03894 (PMC12670633; doi:10.1021/acsanm.5c03894)
Supplement: Supplementary file 1 [file an5c03894_si_001.pdf]

Supporting Information

Optimizing the Graphene/α-Al_2O_3(0001) Interface through Minimization of Interfacial Stress for Improved Electronic Applications

Debdipto Acharya,[a] Daniele Perilli,[a,*] and Cristiana Di Valentin[a]

[a] Department of Materials Science, University of Milano-Bicocca, via R. Cozzi 55, 20125 Milano, Italy

[*] Corresponding author: daniele.perilli@unimib.it

Table S1. Calculated lattice parameters (in Å) and electronic band gaps (in eV) for bulk α-Al_2O_3 using different exchange–correlation functionals, compared with values reported in previous literature.

	Our calculations			From literature[1,2]						
	**PBE**	**HSE06**	**PBE0**	**LDA**	**PBE**	**rPBE**	**PBE_{SOL}**	**PBE0**	**HSE06**	**Exp.**
**a (Å)**	4.79	4.79	4.79	4.71	4.78	4.82	4.75	4.78	4.78	4.75
**c (Å)**	13.06	13.06	13.06	12.85	13.07	13.17	12.96	13.07	13.07	12.95
**E_g(Å)**	6.02	8.62	9.38	6.59	6.05	5.88	5.88	8.55	8.09	8.8

Table S2. Relative energies (E_{rel}, in meV/C) with respect to the most stable configuration (shown in **Figure 2a**), and graphene–alumina vertical distance (in Å) for different stacking configurations of planar R0 (2×2) graphene on the (1×1) Al_2O_3 (0001) surface. Results are reported for both a 12-layer slab with the two middle layers fixed and an 18-layer fully relaxed slab.

Flat R0 Gr	Fig. 2a		Fig. 2b		Fig. 2c		Fig. 2d		Fig. 2e		Fig. 2f	
	**E_{rel} (meV/C)**	**d (Å)**	**E_{rel} (meV/C)**	**d (Å)**	**E_{rel} (meV/C)**	**d (Å)**	**E_{rel} (meV/C)**	**d (Å)**	**E_{rel} (meV/C)**	**d (Å)**	**E_{rel} (meV/C)**	**d (Å)**
12 Al_2O_3 layers	0	2.98	+6	3.12	+2	3.06	+1	3.04	+2	3.06	+1	3.04
18 Al_2O_3 layers	0	3.02	+5	3.16	+1	3.06	+1	3.05	+2	3.08	+3	3.10

Table S3: Relative energies (E_{rel}, in eV) with respect to the most stable configuration (shown in **Figure 4a**) for different stacking configurations of corrugated R0 (6×6) graphene on the (3×3) Al_2O_3 (0001) surface, as shown in **Figure 4**. Calculations were performed using a 12-layer Al_2O_3 slab with the two middle layers fixed. Also reported are the average distance between graphene and the top Al_2O_3 layer (d_{avg}, in Å) and the corrugation amplitude of graphene (Δz, in Å), defined as the height difference (z coordinate) between the highest and lowest C atoms.

**Corrugated R0 (6×6) graphene**	Fig. 4a			Fig. 4b			Fig. 4c		
	**E_{rel} (meV/C)**	**d_{avg} (Å)**	**Δz (Å)**	**E_{rel} (meV/C)**	**d_{avg} (Å)**	**Δz (Å)**	**E_{rel} (meV/C)**	**d_{avg} (Å)**	**Δz (Å)**
12 Al_2O_3 layers	0	3.09	1.32	+6	3.35	1.28	+6	3.19	1.34
	Fig. 4d			Fig. 4e			Fig. 4f		

	E_{rel} (meV/C)	d_{avg} (Å)	Δz (Å)	E_{rel} (meV/C)	d_{avg} (Å)	Δz (Å)	E_{rel} (meV/C)	d_{avg} (Å)	Δz (Å)
	+1	3.12	1.41	+1	3.14	1.13	+1	3.12	1.32

Table S4. Calculated lattice mismatch (%) for various commensurate supercells of Gr on a 12-layer Al-terminated α-Al_2O_3 (0001) surface, obtained by adjusting the Gr lattice to that of Al_2O_3 at different rotational angles (up to 30°). For each angle, the smallest supercell yielding a lattice mismatch below ±1.0% is selected. Positive and negative mismatch values correspond to tensile and compressive strain in Gr, respectively. The table lists the Gr and Al_2O_3 supercells, the resulting periodicity (in nm), and the total number of atoms per supercell, including the 12-layer Al_2O_3 slab.

**Gr rotational angle w.r.t. (1x1) Al_2O_3**	**0°**	**4.7°**	**13.9°**	**19.1°**	**30°**
**Lattice mismatch (%)**	+0.73	-0.09	+0.06	+0.96	-0.31
**Gr supercell**	27 × 27	5√49 × 5√49	7√13 × 7√13	8√7 × 8√7	9√3 × 9√3
**Al_2O_3 (0001) supercell**	14 × 14	18 × 18	13 × 13	11 × 11	8 × 8
**Periodicity (nm)**	6.70	8.62	6.22	5.27	3.83
**No. Atoms (Al_2O_3 + Gr = total)**	1458 + 3920 = 5378	2450 + 6480 = 8930	1274 + 3380 = 4654	896 + 2420 = 3316	486 + 1280 = 1766

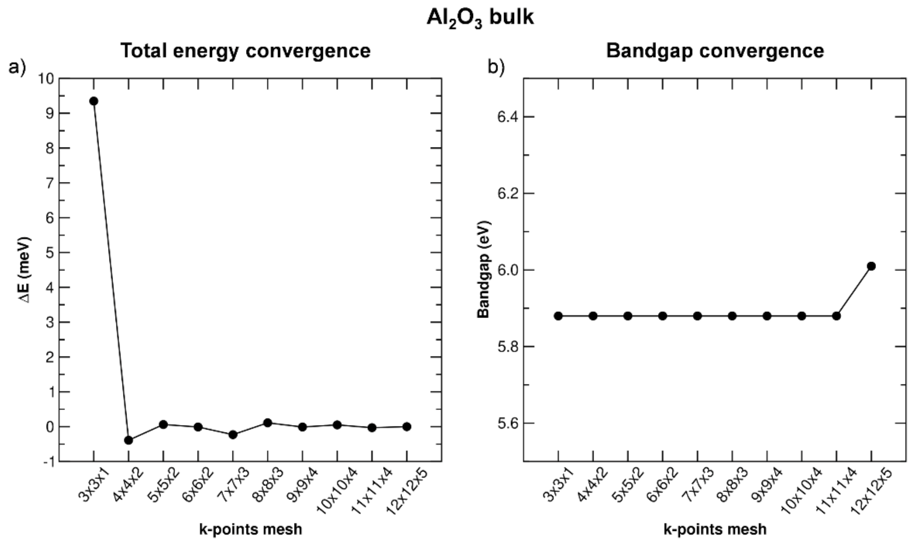

Figure S1. Convergence of (a) the total energy and (b) the electronic band gap with respect to the k-point mesh for the conventional cell of bulk Al_2O_3. Panel (a) shows the total energy difference relative to the finest k-point mesh.

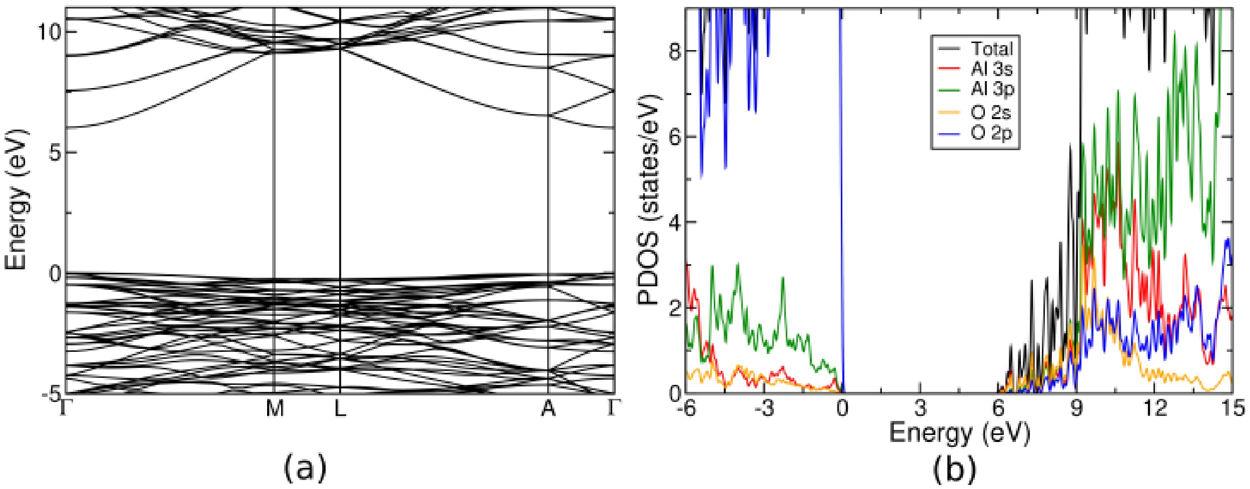

Figure S2. (a) Electronic band structure along high-symmetry directions of the Brillouin zone and (b) projected density of states (PDOS) for bulk α-Al_2O_3. All energy levels are aligned to the top of the valence band, set to 0 eV. The legend for the PDOS is shown in panel (b).

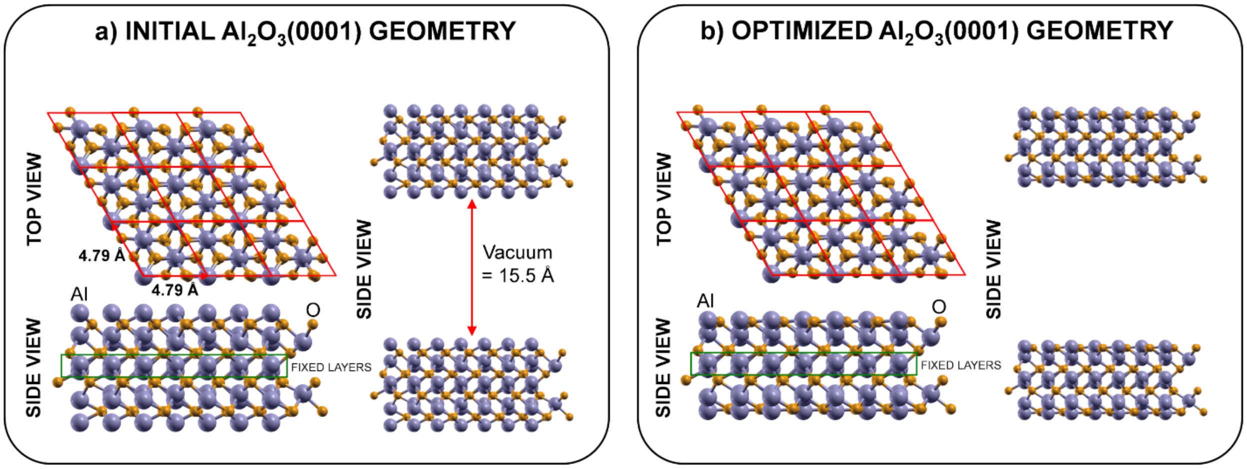

Figure S3. Top and side views of the (a) initial bulk-truncated and (b) optimized $Al_2O_3(0001)$ surface. In the side view, Al and O atoms are indicated, along with the two fixed Al layers. The unit cell is highlighted in red in the top view and is repeated three times along both periodic directions. The vacuum space between the repeated slabs is shown in panel (a), with its thickness indicated in Å. Comparison of the side views before and after relaxation shows the surface relaxation, with contraction of the Al top layer, which in the optimized structure becomes nearly coplanar with the O second layer.

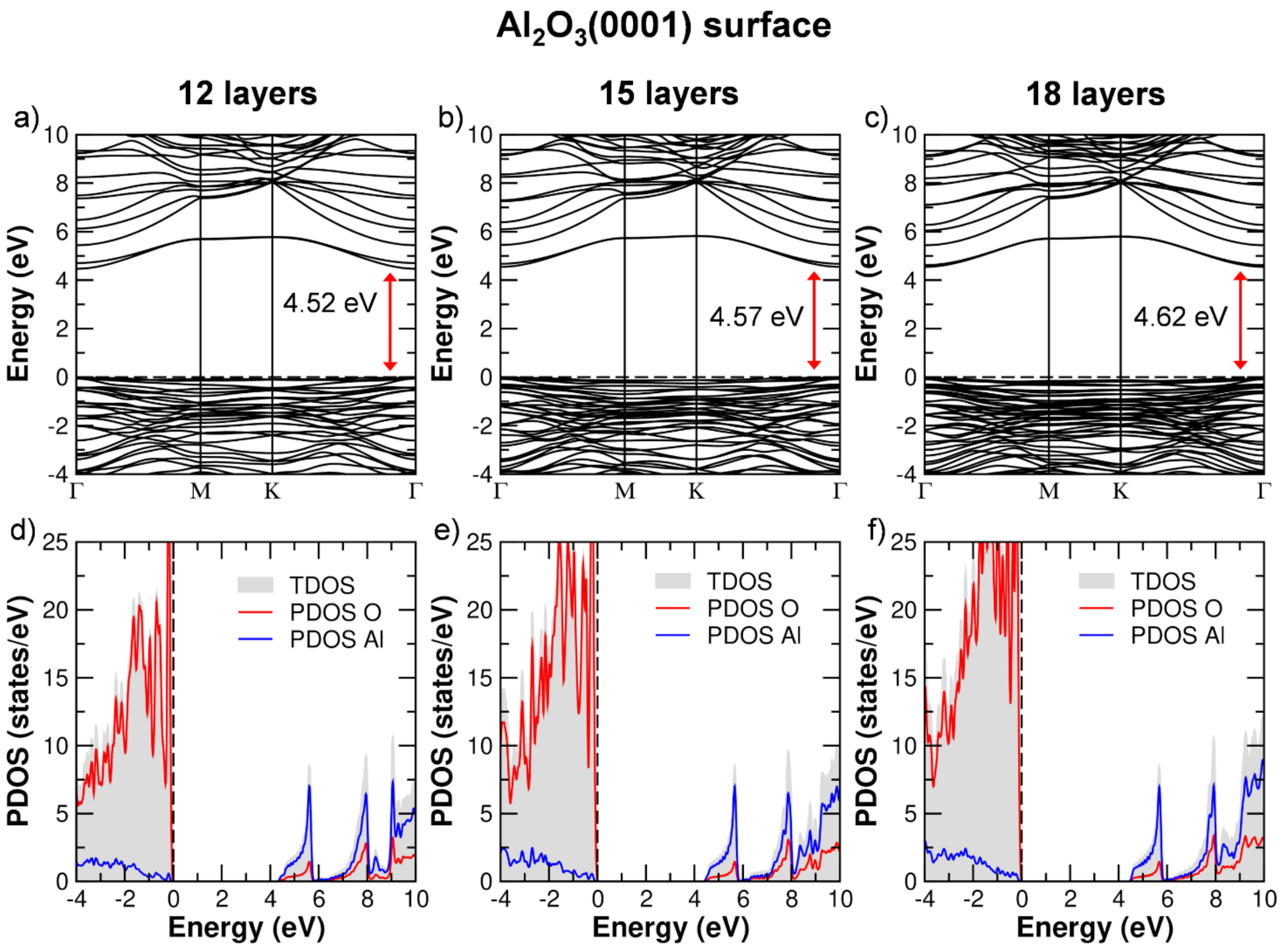

Figure S4. Band structures of the Al_2O_3(0001) surface for (a) a 12-layer slab with the two central Al layers fixed, (b) a fully relaxed 15-layer slab, and (c) a fully relaxed 18-layer slab. Panels (d), (e), and (f) show the corresponding projected density of states (PDOS) for the 12-, 15-, and 18-layer slabs, respectively. PDOS legends are included within each panel. All energy levels are referenced to the valence band maximum, set to 0 eV. The direct band gap at the Γ point is indicated by a red arrow in panels (a)–(c).

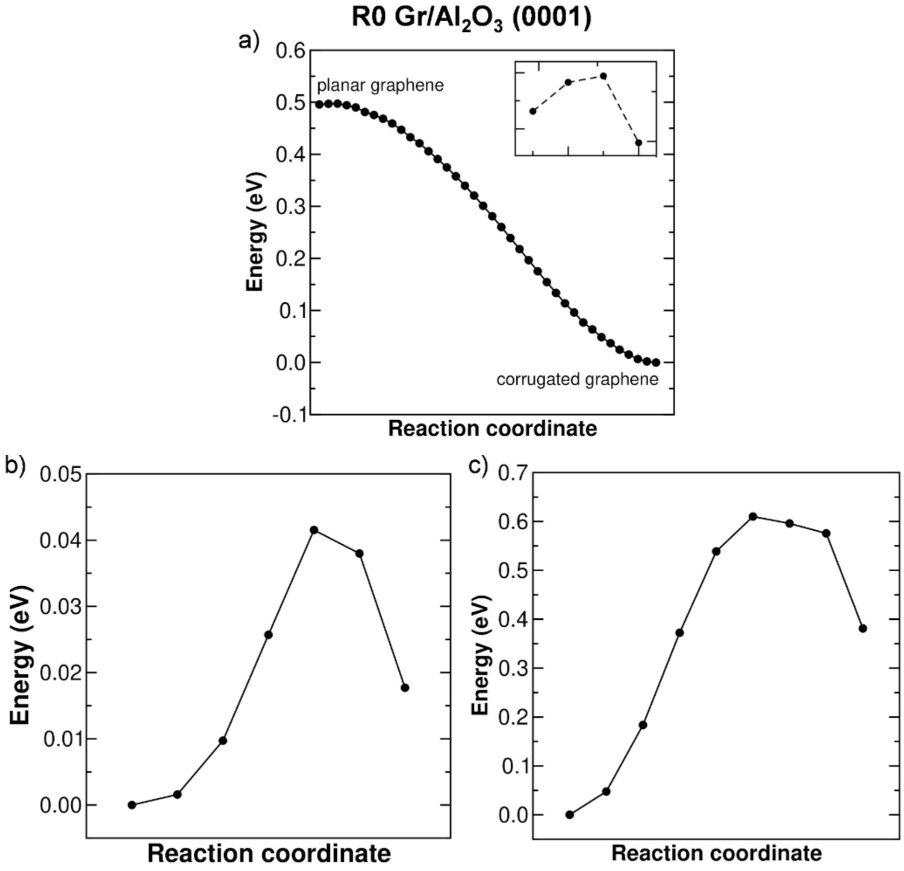

Figure S5. a) Energy profile for the transition from planar to corrugated R0 (6×6) Gr on (3×3) $Al_2O_3(0001)$ surface, calculated using the CI-NEB method. The energy of the corrugated configuration is set to zero. The inset shows a zoom-in of the first few intermediate images, highlighting the presence of a small energy barrier. b) CI-NEB energy profile for in-plane sliding of planar graphene between two configurations (from **Figure 2a** to **2c**). c) CI-NEB energy profile for in-plane sliding of corrugated graphene between two configurations (from **Figure 4a** to **4c**). The energy barrier for corrugated graphene is significantly higher than that observed for planar Gr, indicating that corrugation strongly hinders lateral mobility on the Al_2O_3 surface.

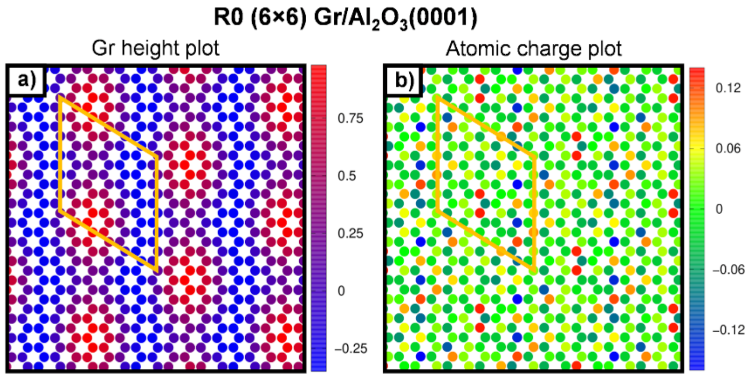

Figure S6. (a) Gr height map (scale in Å) corresponding to **Figure 5a**, and (b) Bader charge distribution (scale in *e*, where positive and negative values indicate electron depletion and accumulation, respectively). The supercell is highlighted in orange. The region containing the geometrically lowest C atoms in panel (a) corresponds to the area of maximum charge accumulation in panel (b). This charge redistribution arises because these C atoms are located above surface Al atoms, thereby maximizing the electrostatic interaction.

References

[1] Rohmann, C., Metson, J. B., & Idriss, H. (2011). DFT study of carbon monoxide adsorption on α-Al2O3 (0001). Surface science, 605(17-18), 1694-1703.

[2] Santos, R. C. R., Longhinotti, E., Freire, V. N., Reimberg, R. B., & Caetano, E. W. S. (2015). Elucidating the high-k insulator α-Al2O3 direct/indirect energy band gap type through density functional theory computations. Chemical Physics Letters, 637, 172-176.
